# Protein inter-domain linker prediction using Random Forest and amino acid physiochemical properties

**DOI:** 10.1186/1471-2105-15-S16-S8

**Published:** 2014-12-08

**Authors:** Maad Shatnawi, Nazar Zaki, Paul D Yoo

**Affiliations:** 1 College of Information Technology, United Arab Emirates University (UAEU), Al-Ain, UAE; 2 Dept. Electrical and Computer Engineering, Khalifa University, Abu Dhabi, UAE

**Keywords:** Protein domain-linker prediction, random forest, machine learning, physiochemical properties, linker index

## Abstract

**Background:**

Protein chains are generally long and consist of multiple domains. Domains are distinct structural units of a protein that can evolve and function independently. The accurate prediction of protein domain linkers and boundaries is often regarded as the initial step of protein tertiary structure and function predictions. Such information not only enhances protein-targeted drug development but also reduces the experimental cost of protein analysis by allowing researchers to work on a set of smaller and independent units. In this study, we propose a novel and accurate domain-linker prediction approach based on protein primary structure information only. We utilize a nature-inspired machine-learning model called Random Forest along with a novel domain-linker profile that contains physiochemical and domain-linker information of amino acid sequences.

**Results:**

The proposed approach was tested on two well-known benchmark protein datasets and achieved 68% sensitivity and 99% precision, which is better than any existing protein domain-linker predictor. Without applying any data balancing technique such as class weighting and data re-sampling, the proposed approach is able to accurately classify inter-domain linkers from highly imbalanced datasets.

**Conclusion:**

Our experimental results prove that the proposed approach is useful for domain-linker identification in highly imbalanced single- and multi-domain proteins.

## Introduction

A domain is a conserved part of a protein that can evolve, function, and exist independently. Each domain forms a three-dimensional (3D) structure and can be folded and stabilized independently. Several domains could be joined together in different combinations forming multi-domain proteins, and perform specific biological task [1,2]. One domain may exist in multiple proteins. A domain varies in length ranging from 25 to 500 amino acids (AAs) [3]. Inter-domain linkers tie neighboring domains and support inter-domain communications in multi-domain proteins. They also provide sufficient flexibility to facilitate domain motions and regulate the inter-domain geometry [4]. Predicting inter-domain linkers is of great importance in precise identification of structural domains within a protein sequence. Many domain prediction approaches first detect domain-linkers, and then predict the location of domain regions accordingly. This domain knowledge is then used to understand protein structures, functions, and evolution, to perform multiple sequence alignment, and to predict protein-protein interactions. In addition, downsizing proteins into functional domains without losing useful biological information leads to significant reduction in the computational cost of protein analysis [5,6]. Therefore, the development of accurate computational method for splitting proteins into structural domains is regarded as a critical step in protein tertiary structure prediction and proteomics [7].

A number of protein inter-domain linker prediction methods have been developed and these methods can be classified into (*i*) statistical-based, (*ii*) alignment/homology-based and (*iii*) machine-learning (ML)-based methods. Dom-Cut [3] is one of the typical early day's statistical-based methods. Domcut predicts inter-domain linker regions based on the differences in AA compositions between domain and linker regions in a protein sequence. DomCut considers a region or segment in a sequence as a linker if it is in the range between 10 and 100 residues, connecting two adjacent domains, and not containing membrane spanning regions. To represent the preference for AA residues in linker regions, it defines the linker index as the ratio of the frequency of AA residue in domain regions to that in linker regions. A linker preference profile is generated by plotting the averaged linker index values along an AA sequence using a siding window of size 15AAs. A linker is predicted if there was a trough in the linker region and the averaged linker index value at the minimum of the trough is lower than the threshold value. At the threshold value of 0.09, the sensitivity and selectivity of DomCut were 53.5% and 50.1%, respectively. Despite the fact that DomCut showed glimpse of potential success, it was reported by Dong *et al*. [8] that DomCut has low sensitivity and specificity compared to other recent methods. However, integrating more biological evidences with the linker index could enhance the prediction and therefore, the idea of DomCut was later utilized by several researchers such as Zaki *et al*. [9] and Pang *et al*. [10].

Shatnawi and Zaki [11] used AA compositional index profile, which combines linker index and AA composition. They divided the protein sequence into chunks and then applied a simulated annealing algorithm to predict the optimal threshold value for each chunk. Linding *et al*. [12] proposed another statistical-based method called GlobPlot. GlobPlot allows users to plot the tendency within protein sequences for exploring both potential globular and disordered/flexible regions in proteins based on their AA sequence, and to identify inter-domain segments containing linear motifs.

A typical alignment/homology-based method which requires the use of PSI-BLAST [13] to generate evolutionary and homology information is DOMpro [14]. DOMpro was independently evaluated along with 12 other predictors in the Critical Assessment of Fully Automated Structure Prediction 4 (CAFASP-4) [15,16] and it was ranked among the top *ab initio *domain predictors. Other popular homology-based methods include Scooby-Domain [17], and FIEFDom [18].

ML-based methods have gained lots of attentions in protein domain-linker prediction tasks. Recent approaches employ machine learning techniques such as Artificial Neural Networks (ANN) and variants of Support Vector Machines (SVM). Sim *et al*. [19] introduced PRODO as an ANN classifier that is trained using features obtained from the position specific scoring matrix (PSSM) generated by PSI-BLAST. The training dataset contained 522 contiguous two-domain proteins was obtained from the structural classification of proteins (SCOP) database, version 1.63 [20]. When tested on 48 newly added non-homologous proteins in SCOP version 1.65 and on CASP5 targets, PPRODO achieved 65.5% of prediction accuracy. ANN models have also used in DomNet [2], DOMpro [14], Shandy [21], and ThreaDom [22].

Ebina *et al*. [23] developed a protein linker predictor called DROP which utilizes a SVM with a Radial Basis Function (RBF) kernel. The classifier is trained using 25 optimal features. The optimal combination of features was selected from a set of 3000 features using a Random Forest (RF) algorithm. The selected features were related to secondary structures and PSSM elements of hydrophilic residues. The accuracy of DROP was evaluated by two domain-linker datasets; DS-All [24,25], and CASP8 FM. DS-All contains 169 protein sequences, with a maximum sequence identity of 28.6%, and 201 linkers. DROP showed a sensitivity and precision of 41.3% and 49.4%, respectively. Varients of SVM have also been used in DomainDiscovery [26], Chatterjee *et al*. [27], and DoBo [28]. The above-mentioned methods, in general, have the following limitations:

• Although methods that use structural information could achieve good prediction results, finding the structural information by itself is another challenge. In contrast, predicting the domain-linkers could lead to infer the structural information.

• ML-based domain predictors have shown limited capability in multi-domain proteins [2].

• Although homology-based methods can achieve high prediction accuracy specially when close templates are retrieved, the accuracy often decreases piercingly when the sequence identity of target and template is low [22].

• Some methods discard any protein sequence with non-contiguous domains. Therefore, domains that are connected by small linkers may not be identifiable.

• Most ML-based methods are computationally expensive. They require the high computational cost to generate PSSM and/or predict secondary structure information for each protein.

• Some methods are evaluated based on the overall prediction accuracy only. This may not effectively reflect the issues of the unbalancing problem of protein domain-linker data.

In this study, we develop a compact and accurate domain-linker prediction approach based solely on protein primary structure information. The novel profile containing AA physiochemical properties and linker indices is trained by a Random Forest (RF) classifier. The linker index is deduced from the protein sequence dataset of domain-linker segments. A sliding window of variable length is used to extract the information on the dependencies of each AA and its neighboring residues. The proposed approach efficiently processes high-dimensional multi-domain protein data with a much more accurate predictive performance than existing state-of-the-art approaches. In our approach, the well-accepted pre-processing techniques causing computational complexity such as class weights or data re-sampling are not used.

## Method

The proposed approach consists of five consecutive steps. First, we construct a multidomain benchmark dataset for fair comparison to the existing developed methods. Second, we build a novel profile that contains useful structural and physiochemical information of protein sequences for the protein domain-linker prediction tasks. Third, a nature inspired ML model using RF is constructed. The ML model is trained by the profile constructed in the second step. Fourth, we find the optimal averaging window size that slides across the protein AA sequence. Last, all of the above steps are integrated and its performance is compared to other existing ML models and domain-linker predictors on two benchmark datasets.

### Datasets

As mentioned, two protein sequence datasets are used to evaluate the performance of our approach. The first dataset is DS-All [24,25] which was used to evaluate DROP [23]. All the sequences in DS-All were extracted from the non-redundant Protein Data Bank (nr-PDB) chain set and contains 182 protein sequences including 216 linker segments. By examining each sequence, we found that the assignment of domains in DS-All dataset is inconsistent with the ones in PDB. We thus relabeled the domains and linkers of the protein sequences of this dataset according to NCBI conserved domains database and ended up with 140 sequences including 334 domains and 183 linker segments. The average numbers of AA residues in linker and domain segments are 12.7 and 147.1 respectively. This means that about 95.5% (334 ×147.1) of the total AA residues are located in domain segments and only 4.5% (183 ×12.7) are in linker segments.

The sequences in the second set were extracted from the Swiss-Prot database [29] and tested by Suyama and Ohara [3] to evaluate the performance of DomCut. This dataset contains 273 non-redundant protein sequences including 486 linkers and 794 domain segments. The average numbers of AA residues in linker and domain segments are 35.8 and 122.1 respectively. Therefore, about 85% (794 × 122.1) of the total AA residues exist in domain segments and only 15% (486 × 35.5) are in linker segments.

### Feature extraction

**Figure 1:**
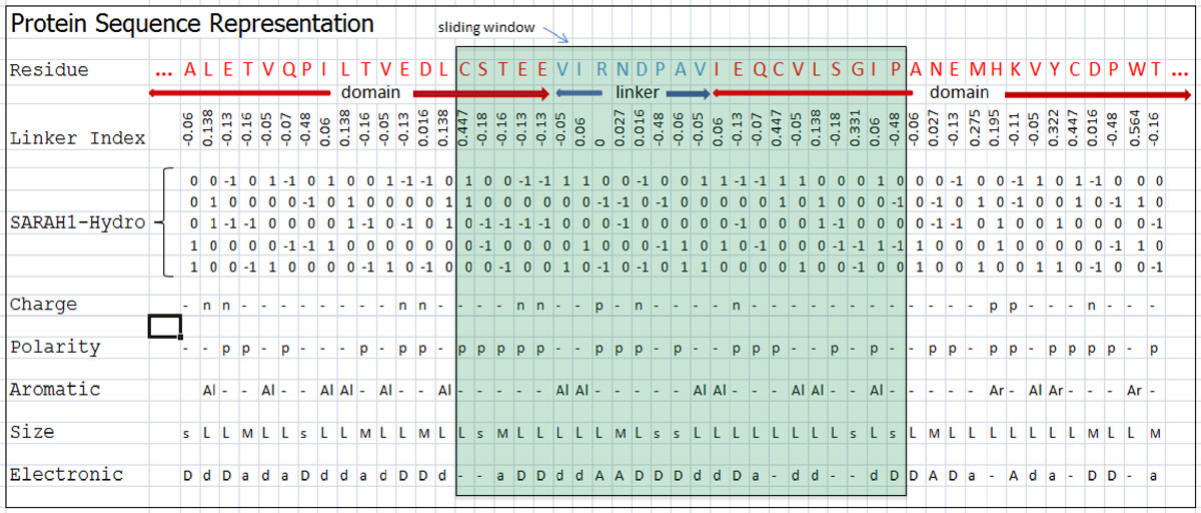
**Representation of proten sequence by AA features and sliding window**. Each sequence in the dataset is replaced by its corresponding properties. These property values are then averaged over a window that slides along the length of each protein sequence.

To extract features from a protein sequence, a sliding window technique is used. For each sequence in the protein dataset, we slide an averaging window across the sequence from the N-terminal to the C-terminal as shown in Figure 1. A number of important features of a protein, located within a sliding window, are extracted. These features include the linker index [3], AA hydrophobicity, and other AA physiochemical properties such as side-chain charge, side-chain polarity, aromaticity, size, and electronic properties.

#### Linker index

Linker index was initially introduced by Suyama and Ohara [3] as a representation of the preference for AA residues in linker regions. Linker index is defined as the ratio of the frequency of AA residue in linker regions to that in domain regions. Linker index *Si *is computed according to the following equation:

(1) $$S_{i}=-ln\left(\frac{f_{i}^{lin\text{ker}}}{f_{i}^{domain}}\right)$$

where $f_{i}^{linker}$ and $f_{i}^{domain}$ are the frequency of AA residue *i *in linker regions and domain regions, respectively. Table 1 shows the frequency of each AA in both linker and domain regions along with its linker index as calculated from DomCut dataset and reported by Suyama and Ohara [3].

**Table 1:** Amino acid composition and linker index.

Amino acid	Linker (%)	Domain (%)	Linker index
P	7.95	4.93	-0.478
S	8.32	6.97	-0.177
T	6.68	5.67	-0.163
E	7.53	6.62	-0.128
K	6.30	5.64	-0.112
Q	4.35	4.04	-0.073
A	7.03	6.64	-0.058
V	7.33	6.96	-0.052
R	5.39	5.39	0.000
D	5.39	5.47	0.016
N	4.29	4.41	0.027
I	4.86	5.16	0.060
L	7.62	8.75	0.138
H	2.13	2.59	0.195
F	2.92	3.71	0.240
M	1.47	1.94	0.275
Y	2.49	3.44	0.322
G	5.46	7.60	0.331
C	1.62	2.53	0.447
W	0.89	1.56	0.564

#### Hydrophobicity profile

Hydrophobic is a physical property of a substance to repel water. Hydrophobicity is a major factor in protein stability. The hydrophobic effect plays a key role in the spontaneous folding of proteins. It can be defined as the free energy required to transfer amino-acid side-chains from cyclohexane to water [30]. Table 2 illustrates hydrophobicity index in kilo-calories per mole for each of the twenty AAs at a pH of 7. Several researchers selected hydrophobicity as the main feature among many other properties in protein structure prediction [30-33], however, it has not been used in detecting domain linkers.

**Table 2:** Hydrophobicity index (kcal/mol) of amino acids in a distribution from non-polar to polar at pH = 7.

Amino acid	Hydrophobicity index	Amino acid	Hydrophobicity index
I	4.92	Y	-0.14
L	4.92	T	-2.57
V	4.04	S	-3.40
P	4.04	H	-4.66
F	2.98	Q	-5.54
M	2.35	K	5.55
W	2.33	N	-6.64
A	1.81	E	-6.81
C	1.28	D	-8.72
G	0.94	R	-14.92

In literature, various hydrophobicity scales have been thoroughly examined for protein sequence classification and prediction tasks. David [34] concluded that the Rose scale was superior to all others when used for protein structure prediction. The Rose scale in Table 3 is correlated to the average area of buried AAs in globular proteins. However, Korenberg *et al*. [32] pointed out several key drawbacks with Rose scale. Since it is not a one-to-one mapping, different amino-acid sequences can have identical hydrophobicity profiles; the scale covers a narrow range of values while causing some AAs to be weighted more heavily than others. To overcome this problems, the SARAH1 scale for AA was introduced [32]. SARAH1 assigns each AA a unique five-bit signed code, where exactly two bits are non-zero. SARAH1 ranks 20 possible AAs according to the Rose hydrophobicity scale. Each AA is assigned a five-bit code in descending order of the binary value of the corresponding code as illustrated in Table 4 where the right-half is the negative mirror image of the left-half. The 10 most hydrophobic residues are positive, and the 10 least hydrophobic residues are negative. In this work, we experimentally tested the three above mentioned AA hydrophobicity scales. SARAH1 scale showed a slightly better prediction accuracy. Thus, we used SARAH1 in the construction of our AA feature set.

**Table 3:** Rose hydrophobicity scale.

Amino acid	Hydrophobicity index	Amino acid	Hydrophobicity index
A	0.74	L	0.85
R	0.64	K	0.52
N	0.63	M	0.85
D	0.62	F	0.88
C	0.91	P	0.64
Q	0.62	S	0.66
E	0.62	T	0.70
G	0.72	W	0.85
H	0.78	Y	0.76
I	0.88	V	0.86

The scale is correlated to the average area of buried AAs in globular proteins.

**Table 4:** SARAH1 hydrophobicity scale.

Amino acid	Hydrophobicity index	Amino acid	Hydrophobicity index
C	1,1,0,0,0	G	0,0,0,-1,-1
F	1,0,1,0,0	T	0,0,-1,0,-1
I	1,0,0,1,0	S	0,0,-1,-1,0
V	1,0,0,0,1	R	0,-1,0,0,-1
L	0,1,1,0,0	P	0,-1,0,-1,0
W	0,1,0,1,0	N	0,-1,-1,0,0
M	0,1,0,0,1	D	-1,0,0,0,-1
H	0,0,1,1,0	Q	-1,0,0,-1,0
Y	0,0,1,0,1	E	-1,0,-1,0,0
A	0,0,0,1,1	K	-1,-1,0,0,0

Each AA is assigned a five-bit code in descending order of the binary value of the corresponding code where the right-half is the negative mirror image of the left-half. The 10 most hydrophobic residues are positive, and the 10 least hydrophobic residues are negative.

#### Physiochemical properties

In addition to hydrophobicity, we considered a few physiochemical properties of AAs as features including electric charge, polarity, aromaticity, size, and electronic property. AAs are categorized according to each physiochemical property as in Table 5[35-37]. Each physiochemical property of an AA is based on its side-chain propensity and has its own characteristics. Physiochemical properties play important roles in recognizing the behavior of AAs and their interactions with other AAs. These interactions have significant impact on the formation, folding, and stabilization of protein 3D structures. For example, polar and charged AAs are able to form hydrogen bonds, and thus, they cover the molecules surfaces and are in contact with solvents. Positively and negatively charged AAs form salt bridges. Polar AAs are hydrophilic, whereas non-polar amino acids are hydrophobic, which are used to twist protein into useful shapes [38,39].

**Table 5:** Amino acid classification according to their physiochemical properties.

Peoperty	Value	Amino acids
Charge	Positive	H, K, R
	Negative	D, E
	Neutral	A, C, F, G, I, L, M, N, P, Q, S, T, V, W, Y

Polatity	Polar	C, D, E, H, K, N, Q, R, S, T, Y
	Non-polar	A, F, G, I, L, M, P, V, W

Aromaticity	Aliphatic	I, L, V
	Aromatic	F, H, W, Y
	Neutral	A, C, D, E, G, K, M, N, P, Q, R, S, T

Size	Small	A, G, P, S
	Medium	D, N, T
	Large	C, E, F, H, I, K, L, M, Q, R, V, W, Y,

Electronic	Strong donor	A, D, E, P
	Weak donor	I, L, V
	Neutral	C, G, H, S, W
	Weak acceptor	F, M, Q, T, Y
	Strong acceptor	K, N, R

### Protein sequence representation

Each sequence in the dataset is replaced by its corresponding properties; linker index, hydrophobicity, charge, polarity, aromaticity, size, and electronic property. These values are then averaged over a window that slides along the length of each protein sequence. To calculate the average feature values $X_{J}^{w}$ along a protein sequence S, using a sliding window of size *w*, we apply the following formula:

(2) $$X_{j}^{w}=\left\{\begin{array}{c} \frac{\sum_{i=1}^{j+\left(\left(w-1\right)/2\right)}x_{si}}{j+(\left(w-1\right)/2};\;\;1\leq j\leq \left(w-1\right)/2\\ \frac{\sum_{i=j-\left(\left(w-1\right)/2\right)}^{j+\left(\left(w-1\right)/2\right)}x_{si}}{j+(\left(w-1\right)/2};\;\;\left(w-1\right)/2<j\leq L-\left(\left(w-1\right)/2\right)\\ \frac{\sum_{i=j}^{L}x_{si}}{L-j+1+\left(\left(w-1\right)/2\right)};\;L-\left(\left(w-1\right)/2\right)<j\leq L \end{array}\right)$$

where *L *is the length of the protein sequence and *x_si _*is the feature vector for the AA residue *s_i _*which is located at position *i *in the protein sequence *S*. Figure 1 depicts the protein sequence representation by the amino acid features and the sliding window.

### Random Forest model

**Figure 2:**
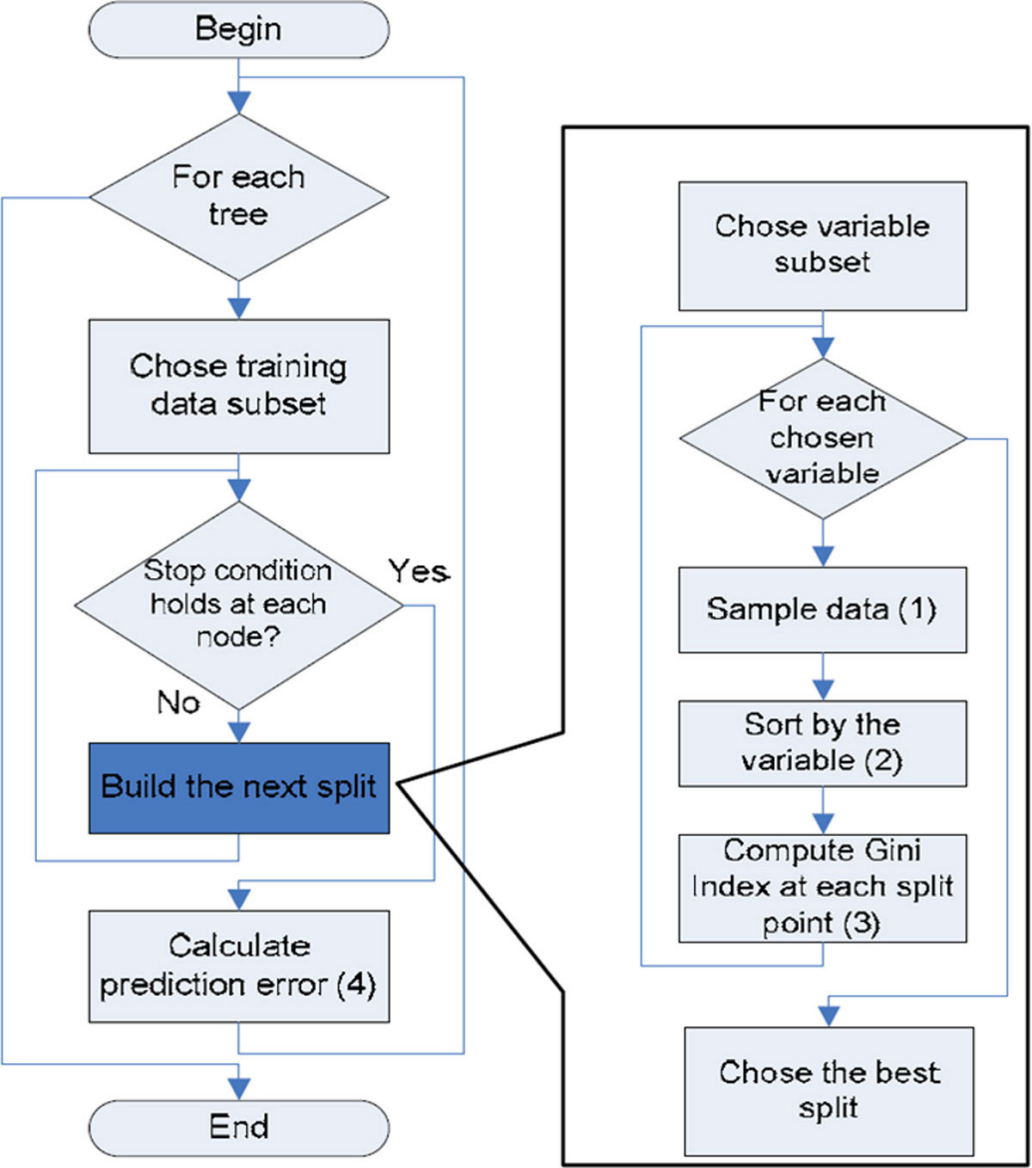
**Random Forest algorithm**.

Random Forest (RF) [40] is an ensemble learner that constructs a multitude of decision trees with randomly selected features during training time and outputs the class that is the mode of the classes output by individual trees. Each decision tree grows as follows: for a training set of *N *cases and *M *variables, sample *n *cases with replacement from the original data to grow the tree. A number *m << M *is specified such that at each node *m *variables are selected randomly to best split the nodes. Each tree grows as large as possible. The error of RF depends on the strength of each individual tree and the correlation between them [41]. RF algorithm is depicted in Figure 2. We set the number of selected features at each node for building the trees, *m*, to (*log*_2_(*number of attributes*) + 1) as recommended by [40]. We examined several values for the number of generated decision trees, *N_trees_*, in the range of 10 and 500 and found that the prediction accuracy increases as *N_trees _*increases. However, the improvement in prediction when *N_trees _*exceeds 200 is not considerable when compared with the increase in computational time and memory. Therefore, we set *N_trees _*to 200 in all the conducted experiments.

Due to its averaging strategy, RF classifier is robust to outliers and noise, avoids overfitting, is relatively fast, simple, easily parallelized, and performs well in many classification problems [40,42]. RF shows a significant performance improvement over the single tree classifiers such as CART and C4.5. RF model interprets the importance of the features using measures such as decrease mean accuracy or *Gini *importance [43]. RF benefit from the randomization of decision tress as they have low-bias and high variance. RF has few parameters to tune and less dependent on tuning parameters [44,45].

Ensemble methods including RF, bagging, and boosting have been increasingly applied to bioinformatics. When compared to bagging and boosting ensemble methods, RF has a unique advantage of using multiple feature subsets which is well suited for high-dimensional data as demonstrated by several bioinformatics studies [46]. Lee et al. [47] compared the ensemble of bagging, boosting and RF using the same experimental settings and found that RF is the most successful one. The experimental results through ten microarray datasets in [48] reported that RF is able to preserve predictive accuracy while yielding smaller gene sets compared to diagonal linear discriminant analysis, kNN, SVM, shrunken centroids (SC), and kNN with feature selection. Other advantages of RF such as robustness to noise, lack of dependence upon tuning parameters, and the computation speed have been verified by [44] in classifying SELDI-TOF proteomic data. Wu et al. [49] compared the ensemble methods of bagging, boosting, and RF to individual classifiers of LDA, quadratic discriminant analysis, kNN, and SVM for MALDI-TOF (matrix assisted laser desorption/ionization with time-of-flight) data classification and reported that among all methods RF gives the lowest error rate with the smallest variance. RF also has better generalization ability than Ababoost ensembles [50].

Recently, RF has been successfully employed to a wide range of bioinformatics problems including protein-protein binding sites [51], protein-protein interaction [52,53], protein disordered regions [54], transmembrane helix [39], residue-residue contact and helix-helix interaction [41], and solvent accessible surface area of TM helix residues in membrane proteins [55].

### Evaluation measures

The most commonly used evaluation metrics in domain-linker prediction tasks are accuracy, recall, precision, and F-measure. Accuracy (Ac) is defined as the proportion of correctly predicted linkers and domains to all of the structure-derived linkers and domains listed in the dataset. Sensitivity, or recall (R), is defined as the proportion of correctly predicted linkers to all of the structure-derived linkers listed in the dataset. Precision (P) is defined as the proportion of correctly predicted linkers to all of the predicted linkers. The F-measure (F1) is an evaluation metric that combines precision and recall into a single value. It is defined as the harmonic mean of precision and recall [56,57]. These four evaluation metrics can be formulated as:

(3) $$Ac=\frac{TP+TN}{TP+TN+FN+FP}$$

(4) $$R=\frac{TP}{TP+FN}$$

(5) $$P=\frac{TP}{TP+FP}$$

(6) $$F1=\frac{2PR}{P+R}$$

where TP (true positive) is the number of AAs within the known linker segment predicted as linkers, TN (true negative) is the number of AAs within the known domain segment predicted as domains, FN (false negative) is the number of AA within the known linker segments predicted as domains, and FP (false positive) is the number of AA within the known domain segment predicted as linkers.

Recall and precision are useful measures in domain-linker prediction problem. Recall and precision are class-independent measures so that they can handle unbalanced data situation, where data points are not equally distributed among classes such as domain-linker data. F1-score is also used as a unified measure to compare two approaches when one approach has higher recall and lower precision than the other.

## Results

**Figure 3:**
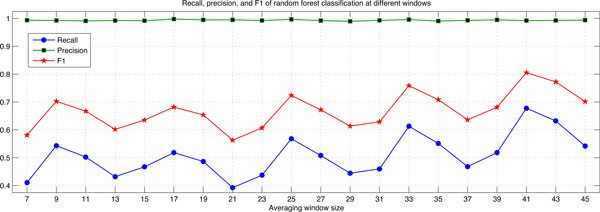
**Averaging window optimization**. Recall, precision, and F1-score at different window sizes with fifty protein sequences from DS-All dataset.

**Figure 4:**
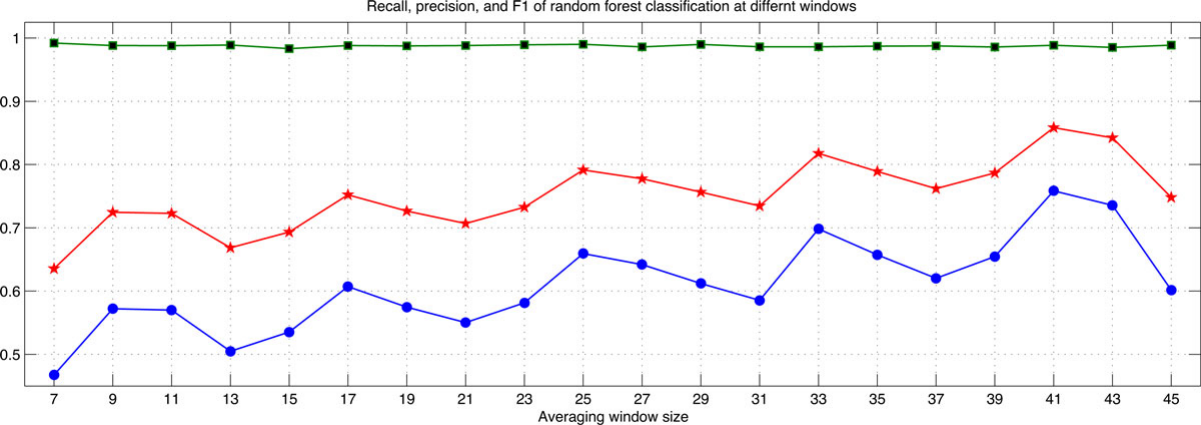
**Averaging window optimization**. Recall, precision, and F1-score at different window sizes with fifty protein sequences from DomCut dataset.

To find the optimal averaging window size, we tested odd window sizes in the range of 7 to 45 residues at randomly selected 50 protein sequences from DS-All dataset [23] and another randomly selected 50 protein sequences from DomCut dataset [3], and then compared the prediction performance at these windows in terms of recall, precision, and F1-score. Figure 3 depicts the performance measures at different sliding windows when applied to the 50 protein sequences of DS-All dataset. Figure 4 shows these prediction measures at different sliding windows when applied to the 50 protein sequences from DomCut dataset. As seen in these two figures, the window size of 41 showed the highest recall, precision and F-measure on both datasets. We thus set the averaging window size to 41 to obtain the final experimental results.

**Figure 5:**
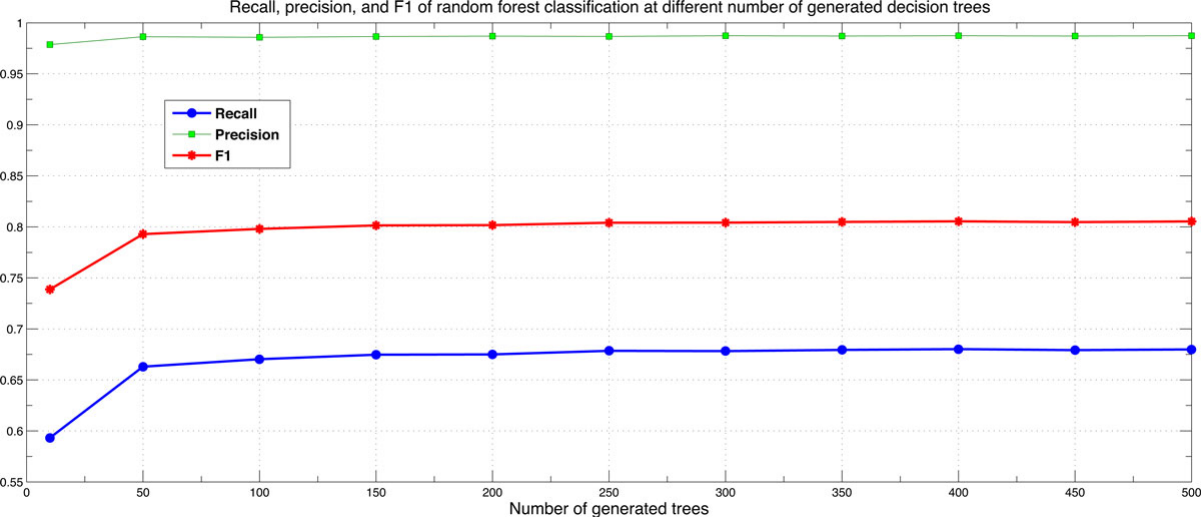
**Number of generated trees optimization**. Recall, precision, and F-measure at different number of generated trees performed on DS-All dataset.

We set the number of selected features at each node for building the trees, *m*, to (*log*2(*number of attributes*) + 1) as recommended by [40]. We examined several values for the number of generated decision trees, *N_trees_*, in the range of 10 and 500 and found that the prediction accuracy increases as *N_trees _*increases as shown in Figure 5. However, the improvement in prediction when *N_trees _*exceeds 200 is not considerable when compared with the increase in computational time and memory. Therefore, we set *N_trees _*to 200 in all the conducted experiments. This also agrees with recent empirical studies [58,59] which reported that ensembles of size less or equal to 100 are too small for approximating the infinite ensemble prediction.

**Figure 6:**
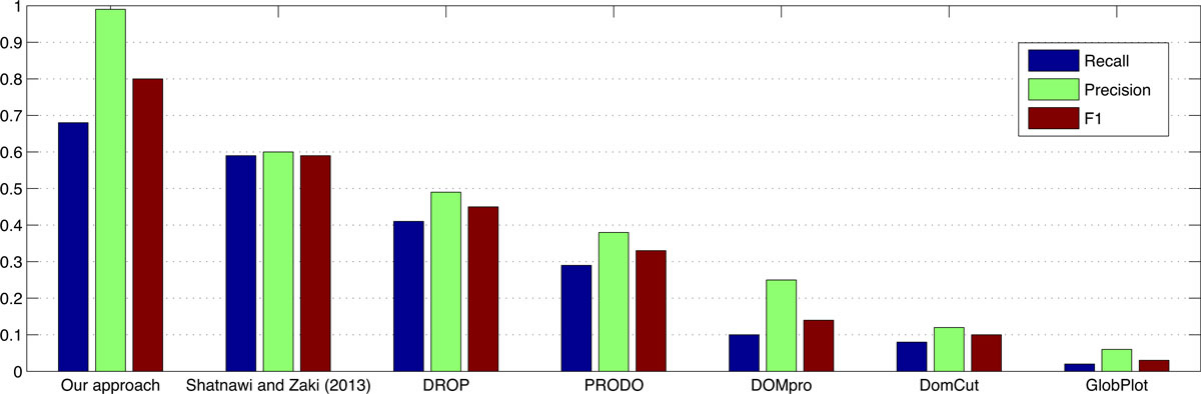
**Performance comparison**. Recall, precision, and F-measure of six currently available domain boundary/linker predictors compared to our approach performed on DS-All dataset.

**Figure 7:**
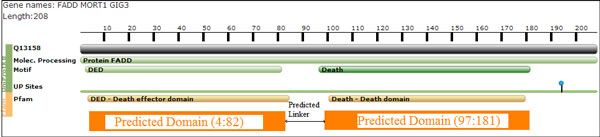
**FADD Human-protein**. The protein chain contains 208 residues and has two domains and a linker in the interval (83-96).

On the DS-All dataset, with 10-fold cross validation, we achieved the average prediction recall of 0.68, precision of 0.99, and F-measure of 0.80. The comparisons of our approach with existing domain-linker predictors approaches [23] on DS-All dataset are summarized in Figure 6. Clearly, the proposed approach outperformed the existing domain-linker predictors in terms of recall, precision, and F-measure. To prove the usefulness of our approach, it was again tested on DomCut/Swiss-Prot protein sequence dataset. Our approach again outperformed Shatnawi and Zaki's predictor [11] as well as DomCut [3] with average recall of 0.65, a precision of 0.98, and an F-measure of 0.78 as shown in Table 6. Figure. 7 shows the predicted domain-linkers by the proposed approach on FAS-associated death domain protein, FADD Human, (PDB Accession number Q13158) which has 208 residues with two domains and one domain-linker located in the interval between 83 and 96 residues.

**Table 6:** Recall, precision, and F-measure using Swiss-Prot/DomCut dataset.

Approach	Recall	Precision	F1
Our Approach	0.71	0.98	0.82
Shatnawi and Zaki [11]	0.56	0.84	0.67
DomCut [3]	0.54	0.50	0.52

## Discussion

The experimental results showed that the proposed approach is useful for the domain-linker identification of highly imbalanced single-domain and multi-domain proteins. There are several advantages of the proposed approach. First, the better predictive performance of the proposed approach was achieved on the imbalance domain-linkers without applying any class weights or data re-sampling techniques. In other words, the proposed approach it is not biased towards the majority class like most other models. To compare RF performance to SVM and ANN, we trained SVM and ANN classifiers with the same protein data and found that both classifiers classified the whole protein sequences as domains. This can be explained by the fact that the training of such methods is based on optimizing the model parameters to maximize the classification accuracy (by minimizing the error rate) which is not a successful strategy in case of highly imbalanced data. Second, physiochemical properties that are used in this approach play important roles in forming the behavior of AAs and their interactions with other AAs and these interactions have significant impact on the formation, folding, and stabilization of protein 3D structures. Therefore, these properties are important features to distinguish structural domains from linkers. Third, AA features that are used in this approach can be extracted with a low computational cost when compared to extracting other features such as PSSM and protein secondary structure that are used in most of the current approaches. Generating PSSM and predicting secondary structure features are computationally expensive and time consuming. Moreover, protein secondary structures are normally predicted by computational methods, and therefore, domain-linker prediction is influenced by secondary structure prediction accuracy as the incorrectly predicted secondary structures may lead to model misclassification.

On the other hand, one of the limitations of our approach is that RF may break the correlation between AAs. Each instance in the training data is a the average feature values for a certain AA residue in the protein. The preference of each AA to exist in domain or linker strongly depends on its neighbor AAs. Therefore, there is a strong correlation between these AA instances and when RF algorithm randomly selects a number of instances for each decision tree, the sequence-order knowledge may be lost.

To find which features contribute most to the prediction, we perform a feature selection procedure as follows. First, we measure the Information Gain (IG) of each feature and order the features according to their IG. Then, we remove the features one by one starting with the one that has least IG and find its effect on the prediction and present the results in Table 7. It is found that AA linker index and hydrophobicity contribute more while AA polarity and electric charge contributes less than other features.

**Table 7:** Prediction measures after removing features that have less information gain using DS-All dataset.

Features Removed	Recall	Precision	F1
None	0.675	0.987	0.802
Polarity	0.673	0.984	0.799
Charge and Polarity	0.645	0.983	0.779
Size and all the above	0.602	0.980	0.746
Electronic and all the above	0.455	0.968	0.619
Aromaticity and all the above	0.325	0.916	0.480
Hydrophobicity and all the above	0.169	0.204	0.185

Although various ML-based domain prediction approaches have been developed, they have shown a limited capability in multi-domain protein prediction. Capturing long-term AA dependencies and developing a more suitable representation of protein sequence profiles that includes evolutionary information may lead to better model performance. Existing approaches showed a limited capability in exploiting long-range interactions that exist among amino acids and participate in the formation of protein secondary structure. Residues can be adjacent in 3D space while located far apart in the AA sequence. [2,60]. Regarding protein sequence profile representation, the proposed input profiles in most domain-linker predictors still provides insufficient structural information to reach the maximum accuracy.

One reason behind the limited capability of multi-domain protein predictors is the disagreement of domain assignment within different protein databases. The agreement between domain databases covers about 80% of single domain proteins and about 66% of multi-domain proteins only [61]. This disagreement is due to the variance in the experimental methods used in domain assignment. The most predominant techniques used to experimentally determine protein 3D structures are X-ray crystallography and nuclear magnetic resonance spectroscopy (NMR). However, their conformational results of domain assignment vary in about 20% so that upper limit accuracy for such domain-linker prediction task could be about 80%.

## Conclusions

To the best of our knowledge, it is clearly novel that the use of well-optimized RF classifier along with a profile that contains domain-linker and physiochemical property information for protein domain linker identification problem. The profile also uses a sliding window of variable length to extract the information on the dependencies of each AA and its neighbors. The utility of the proposed approach is proved on two well-known benchmark datasets by achieving a recall of 68%, precision of 99%, and F1-score of 80%. The proposed approach successfully eliminates some of the data pre-processing steps such as class weights or data re-sampling techniques, and proves that the model can handle imbalanced data and is not biased towards the majority class. This work can be extended by examining longer averaging window sizes in order to capture long-range AA dependency information. The averaging window formula can also be improved to a weighted average so that the closer AA neighbors to the central residue can take higher weights than farther ones.

## Competing interests

The authors declare that they have no competing interests.

## Authors' contributions

MS, NZ, and PY have contributed to the conceptual development of the method. MS has performed the experimental work and the statistical analysis. MS drafted the manuscript. PY and NZ revised it.

## Declarations

The publication cost for this article was funded by the College of Information Technology at the United Arab Emirates University.

This article has been published as part of *BMC Bioinformatics *Volume 15 Supplement 16, 2014: Thirteenth International Conference on Bioinformatics (InCoB2014): Bioinformatics. The full contents of the supplement are available online at http://www.biomedcentral.com/bmcbioinformatics/supplements/15/S16.
